# The presence of CXCR4^+^ CD1a^+^ cells at onset of Langerhans cell histiocytosis is associated with a less favorable outcome

**DOI:** 10.1080/2162402X.2015.1084463

**Published:** 2015-08-31

**Authors:** Willemijn T. Quispel, Janine A. Stegehuis-Kamp, Laura Blijleven, Susy J. Santos, Magda Lourda, Cor van den Bos, Astrid G.S. van Halteren, R. Maarten Egeler

**Affiliations:** a Immunology Laboratory, Willem Alexander Children's Hospital/Leiden University Medical Center, Leiden, the Netherlands; b Childhood Cancer Research Unit, Department of Women's and Children's Health, Karolinska Institutet, Karolinska University Hospital, Stockholm, Sweden; c Center for Infectious Medicine, Department of Medicine, Karolinska Institutet, Karolinska University Hospital, Stockholm, Sweden; d Department of Pediatric Oncology; Emma Children's Hospital/Academic Medical Center, Amsterdam, the Netherlands; e Division of Hematology/Oncology, Hospital for Sick Children/University of Toronto, Toronto, Canada

**Keywords:** Chemokine receptor CXCR4, CXCL12, chemotaxis, Langerhans cell histiocytosis, prognostic marker

## Abstract

Purpose: Langerhans Cell Histiocytosis (LCH) is a neoplastic disorder characterized by tissue accumulating CD1a^+^ histiocytes which frequently carry somatic mutations. Irrespective of mutation status, these LCH-cells display constitutively active kinases belonging to the MAPK pathway. We evaluated, in retrospect, the contribution of individual components of the MAPK-activating and chemotaxis-promoting TNF-CXCR4-CXCL12 axis to LCH manifestation and outcome. Experimental design: CXCR4, CXCL12 and TNF protein expression was immunohistochemically analyzed in 70 LCH-affected biopsies. The presence of CXCR4^+^CD1a^+^ cells in peripheral blood (PB) and/or bone marrow (BM) samples was evaluated by flowcytometry in 13 therapy-naive LCH-patients. Results: CXCL12 was detected in 68/70 (97%) biopsies. CXCR4^+^LCH-cells were present in 50/70 (71%) biopsies; their presence was associated with higher levels of intralesional TNF. Circulating CD1a^+^CXCR4^+^ cells were detected in 4/13 (31%) therapy-naïve LCH-patients which displayed BRAF^V600E^ (2/4), MAP2K1 (1/4) or no (1/4) mutations in their tissues. These CD11c co-expressing CD1a^+^CXCR4^+^cells migrated to CXCL12 in chemotaxis assays. Lesional CXCR4^+^LCH-cells were detected in 18/20 cases who presented with LCH manifestation at multiple sites and in 5/23 (22%) patients who developed additional lesions after initially presenting with a single lesion. The CXCR4 status at onset proved to be an independent risk factor for LCH reactivation in multivariate analysis (odds ratio 10.4, *p* = 0.034). Conclusions: This study provides the first evidence that CXCR4 is involved in the homing and retention of LCH-cells in CXCL12-expressing tissues and qualifies CXCR4 as a candidate prognostic marker for less favorable disease outcome.

## Abbreviations

BM: Bone Marrow
BMMC: Bone Marrow Mononuclear Cells
CHT: chemotherapy
CNS: Central Nervous System
DI: diabetes Insipidus
FFPE: formalin-fixed-paraffin-embedded
IVIG: intravenous immunoglobulin
LC: Langerhans Cell
LCH: Langerhans Cell Histiocytosis
LN: lymph nodes
MAPK-pathway: RAS-RAF-MEK-ERK-pathway
MO: mono ostotic
MS: multi system
PB: Peripheral Blood
PBMC: Peripheral Blood Mononuclear Cells
PO: poly ostotic
PS: penicillin/streptomycin
RTX: radiotherapy
ST: steroids
RE: resection

## Introduction

LCH is characterized by chronically inflamed tissue lesions which contain clonal Langerin^+^ (CD207+)/CD1a^+^ Langerhans Cell (LC)-like histiocytes of myeloid origin (LCH-cells) intermixed with other inflammatory cells. Its clinical manifestation varies from a single site lesion to multiple lesions. The presence of multiple lesions may be limited to the bone (referred to as poly-ostotic LCH) or as part of a multi-system disease. These lesions are either located in ‘non-risk’ organs (bone, lung, gastrointestinal tract, lymph nodes (LN) and skin) or in ‘risk organs’ that is BM, liver and spleen. Its manifestation form, the involvement of risk organs and the patient's clinical response to first line treatment evaluated at 6–12 weeks collectively predict the risk for mortality and treatment failure.^1^ In addition, patients who suffer from multi-system disease and/or multiple reactivations are more likely to develop long-term permanent sequelae than patients with single-system disease.^2,3^

Results from international studies have shown that prolongation of chemotherapy improves reactivation-free survival in multi-system LCH patients.^4,5^ Given that a substantial proportion (∼70%) of multi-system and poly-ostotic patients never reactivates, biomarkers identifying this subcategory of patients at the time of diagnosis are needed as prolonged treatment may lead to an unnecessary burden for both the patient and the health care system. To date, few biological risk markers have been identified which seem useful to predict the development of chronic disease, reactivations or sequelae.^6-9^

In the majority of patients suffering from this rare disease, distinct somatic mutations are detected in lesional LCH-cells. The somatic mutations identified to date are all confined to genes encoding intracellular signaling proteins (kinases) of the RAS-RAF-MEK-ERK (MAPK)-pathway.^10–12^ The cancer-associated BRAF^V600E^ mutation is by far the most frequently detected mutation in LCH-cells followed by MAP2K1 mutations.^10-12^ The impact of these mutations on primary LCH manifestation and long-term outcome is still not clear.^6,13–16^ While genetic screening of LCH-cells present in biopsied tissues may be helpful in the upfront identification of patients who could apply for targeted pharmacotherapy such as RAF or MEK inhibitors,^17^ there are currently no risk markers available for treatment decisions for patients in whom any of these mutations are not found. Given that constitutive expression of active kinases like phosphorylated-ERK have also been detected in the latter category of LCH patients,^13^ there are probably additional mechanisms contributing to ERK activation.

Interactions of chemokines and their receptors play an important role in migration of immune cells and cancer cells to distinct tissues throughout the body. We earlier reported data supporting the concept that LCH-cells fail to upregulate CCR7 and downregulate CCR6. This aberrant chemokine receptor profile could explain their apparent disability to migrate from tissues where the CCR6-binding ligand CCL20 is expressed to regional LN.^18,19^ Histiocytes in other non-LC-histiocytic diseases, such as Erdheim-Chester Disease (ECD) and Rosai-Dorfman disease, also display aberrant co-expression of CCR6 and CCR7.^19,20^ For conventional human LC residing in the upper layers of the skin and mucosa, upregulation of the chemokine receptor CXCR4 is required for subsequent migration to deeper layers of the skin and LN where its sole ligand (Stromal-Derived-Factor-1 or CXCL12) is produced.^21^ Other typical LCH manifestation sites such as bone (marrow), LN and lung all normally express CXCL12.^22^ Furthermore, binding of extracellular CXCL12 to CXCR4 results in the activation of, among others, ERK1/2 proteins,^23^ which could well fit with the observed constitutive expression of phosphorylated-ERK in LCH-cells.^13^ Aside from the inflammation-induced expression of CXCR4 by TNF,^21,24^ CXCR4 (over)expression is associated with the extent and site of metastases as well as with poor prognosis in more than 23 types of cancer.^22,25,26^ Given the neoplastic features of LCH-cells, we assessed whether the TNF-CXCR4-CXCL12 axis is involved in orchestrating the accumulation of LCH-cells at tissue sites where CXCL12 is normally produced. Furthermore, we evaluated CXCR4 expression in relation to LCH manifestation and long-term outcome.

## Materials and methods

### Patient materials

A total of 70 formalin-fixed-paraffin-embedded (FFPE) archived biopsies (bone n = 47, skin n = 12, lung n = 6 and LN n = 5) derived from 57 LCH-patients were obtained at diagnosis (n = 66) or after reactivation (n = 4). Patient characteristics are shown in Table 1. LCH diagnosis was confirmed by clinical symptoms in combination with the presence of CD1a^+^ histiocytes present in biopsied tissues. All CD1a^+^ LCH-cells present in the included biopsies co-expressed Langerin (CD207) so that either marker can be used for visualization of LCH-cells *in situ* (data not shown). PB and/or BM samples were collected from 13 LCH patients at different time points as indicated in the figure legends; buffy coats from whole blood donations by healthy volunteer donors served as controls (Sanquin Blood Supply Foundation, Leiden, The Netherlands). All LCH-patients, and their parents in the case of patients below the age of 18 years, provided verbal or written consent which was registered in the patients’ files and in informed consent forms. Patient characteristics are shown in Table 2. This study was approved by the Medical Ethical Committees of the Leiden University Medical Center (P10.163) and of the Amsterdam Medical Center (METC2013_266). The study was performed according to the guidelines of the national organization of scientific societies (FEDERA).

**Table 1.** Clinical characteristics of LCH patients analyzed for *in situ* CXCR4 and Langerin co expression.

Variable	Total cohort (n = 57 )	Mono-ostotic (n = 26 )	Poly-ostotic (n = 10 )	Multi-system (n = 10 )	LN or skin Single site (n = 7 )	Pulmonary (n = 4 )
Sex, n (%)						
Female	16 (30)	7 (28)	8 (80)	6 (60)	4 (80)	1 (33)
Male	37 (70)	18 (72)	2 (20)	4 (40)	1 (20)	2 (66)
N.A.	4	1	0	0	2	1
Age at onset, n, median (range) in years						
< 18 years	41, 5 (0–17)	21, 7 (0–17)	8, 3 (0–10)	8, 2 (0–17)	3, 10 (0–16)	1, 17 (17)
≥ 18 years	12, 29 (23–65)	4, 27 (23–53)	2, 28 (27–29)	2, 56 (48–65)	2, 25 (23–27)	2, 37 (30–44)
N.A.	4	1	0	0	2	1
CXCR4 protein expression, n (%)						
CXCR4^+^ LCH cells	41 (72)	19 (73)	9 (90)	9 (90)	3 (43)	1 (25)
CXCR4^-^ LCH cells	16 (28)	7 (27)	1 (10)	1 (10)	4 (57)	3 (75)
Mutation status, n (%)						
BRAF WT	20 (60)	7 (64)*	4 (57)	2 (29)**	4 (80)	3 (100)
BRAF V600E mutation	13 (40)	4 (36)	3 (43)	5 (71)	1 (20)	0 (0)
N.A.	24	15	3	3	2	1
Treatment, n (%)						
None	5 (11)	3 (13)	0	0	1 (50)	1 (100)
Resection	2 (5)	1 (4)	0	0	1 (50)	0
Intralesional corticosteroid infiltration	16 (36)	15 (65)	0	1 (11)	0	0
Intralesional corticosteroid infiltration + other	3 (7)	2 (9)	1 (11)	0	0	0
Systemic steroids + chemotherapy	14 (32)	2 (9)	7 (78)	5 (56)	0	0
Systemic steroids + chemotherapy + other	4 (9)	0	1 (11)	3 (33)	0	0
N.A.	13	3	1	1	5	3

Other: either resection or radiotherapy

*One patient displayed the ARAF mutation^10^

**One patient displayed the MAP2K1 mutation^11^

**Table 2.** Clinical characteristics of LCH patients analyzed in parallel for circulating CD1a^+^CD11c^+^CD14^+^CXCR4^+^ cells and *in situ* CXCR4 and Langerin co expression.

LCH	% CD1a^+^ CD11c^+^ CD14^+^ *	CXCR4^+^ LCH-cells	Specimen	Manifestation at diagnosis	Manifestation during follow-up	Development second lesion (**)	Total Follow-up period **	Age of onset ***	Treatment primary lesion	BRAF mutation status	Mutation analysis
1	0	N.A.	PB^a t=-4^	CNS, DI	CNS, DI	No	16	16	ST + CHT	N.A.	N.A.
2	0	N.A.	PB^a t=0^	MO	MO	No	51	7	ST	N.A.	N.A.
3	N.A.	Present	Bone^a t=0^	MO	MO	No	35	7	(local) ST + CHT	ARAF	WES^$^
3	0	N.A.	PB^a t=0^	MO	MO	No	35	7	ST + CHT	N.A.	N.A.
3	0	N.A.	PB^c t=2^	MO	MO	No	35	7	ST + CHT	N.A.	N.A.
3	0	N.A.	BM^b t=11^	MO	MO	No	35	7	local ST	N.A.	N.A.
4	0	N.A.	PB^a t=0^	MO	MO	No	21	4	ST + CHT	N.A.	N.A.
4	0	N.A.	Bone^a t=0^	MO	MO	No	21	4	ST + CHT	V600E	PCR^#^
5	0	N.A.	PB^a t=0^	MO	MO	No	34	4	None	N.A.	N.A.
5	0	N.A.	PB^c t=34^	MO	MO	No	34	4	None	N.A.	N.A.
6	0,01	N.A.	PB^a t=0^	MO	MO	No	4	1	ST	N.A.^∼^	PCR^>^
7	N.A.	Present	Skin^a t=0^	MS	MS	No	112	1	ST + CHT	Wild type	IHC^
7	0,6	N.A.	PB^a t=0^	MS	MS	No	112	1	ST + CHT	N.A.	N.A.
7	0	N.A.	PB^c t=73^	MS	MS	No	112	1	ST + CHT	N.A.	N.A.
7	0,1	N.A.	BM^a t=0^	MS	MS	No	112	1	ST + CHT	N.A.	N.A.
8	N.A.	Present	Skin^a t=0^	MS	MS	No	55	0	ST + CHT	MAP2K1	PCR^$^
8	0	N.A.	PB^b t=8^	MS	MS	No	55	0	ST + CHT	N.A.	N.A.
8	0,2	N.A.	BM^a t=0^	MS	MS	No	55	0	ST + CHT	Wid type	PCR#
9	0,04	N.A.	BM^a t=0^	MS	MS	No	2	1	ST + CHT	V600E	PCRand
9	0	N.A.	PB^a t=0^	MS	MS	No	2	1	ST + CHT	V600E	PCRand
9	N.A.	Present	Skin^a t=0^	MS	MS	No	2	1	ST + CHT	V600E	PCR >
9	N.A.	Present	Subcutis^a t=0^	MS	MS	No	2	1	ST + CHT	N.A.	N.A.
9	N.A.	Present	Mandibula^a t=0^	MS	MS	No	2	1	ST + CHT	N.A.	N.A.
10	0	N.A.	PB^d t=9^	PO	MS	yes (8)	45	4	ST + CHT	N.A.	N.A.
10	0	N.A.	PB^c t=29^	PO	MS	yes (8)	45	4	ST + CHT	N.A.	N.A.
10	0	Present	Bone^a t=0^	PO	MS	yes (8)	45	4	ST + CHT	V600E	PCR$
10	0	N.A.	BM^d t=8^	PO	MS	yes (8)	45	4	ST + CHT	N.A.	N.A.
11	0	N.A.	PB^a t=0^	PO	PO	No	105	10	ST + CHT	N.A.	N.A.
11	0	N.A.	Bone^a t=0^	PO	PO	No	105	10	ST + CHT	Wid type	PCR#
12	0	N.A.	PB^a t=1^	PO	PO	No	10	5	ST + CHT	N.A.	N.A.
13	0	N.A.	PB^a t=1^	PO	PO	No	16	3	None	N.A.	N.A.

Follow up data were available for 13 patients and varied from 65 d to 10 y after primary diagnosis, with a median follow-up time of 45 mo. For patients who developed a second lesion at another site than the (successfully) treated primary lesion, LCH manifestation was designated as multi-site LCH (either poly-ostotic or multi system). LCH indicates Langerhans Cell Histiocytosis; N.A. not available; PB peripheral blood and BM Bone marrow; Diagnosis is referred to as multi system (MS); Mono Ostotic (MO); Poly Ostotic (PO); Diabetes Insipidus or Central Nervous System (CNS, DI); IVIG: Intravenous immunoglobulin ST: Steroids; CHT: chemotherapy;. Samples were collected at different time points after diagnosis (t = x);

^a^sample collected at diagnosis and before treatment initiation;

^b^sample collected during systemic treatment;

^c^sample collected after LCH recovery;

^d^sample collected shortly after the appearance of a second lesion other than the primary LCH lesion; Please note that, in some cases, analysis was performed on multiple samples collected at different time points from the same patient.

*of HLA-DR^+^/CD3^−^/CD20^−^/CD56^−^,

**follow-up data in months from diagnosis until last clinical visit or time between diagnosis and secondary lesion;

***age of onset in years; ∼ LCH-cells in this biopsy expressed BRAF^V600E^; BRAF mutation analysis was assessed by immunohistochemistry (IHC) or by molecular biological techniques, real-time PCR (PCR), either according to standard validated pathology protocols at the AMC (>)^29^; at the University of Heidelberg (^)^15^ or according to protocols developed in research laboratories at Dana Farber Cancer Institute (including pyrosequencing and Whole Exome Sequencing (WES), $)^13^ in the LUMC (#)^34^, or at Sanquin Research (&).

### Immunohistochemistry, immunofluorescent staining and BRAF mutation analysis

4μm FFPE sections were dewaxed and rehydrated as described earlier.^27^ For immunohistochemistry analysis, endogenous peroxidase was blocked by methanol containing 0.3% H_2_O_2_ and antigen retrieval was performed in boiling EDTA buffer pH8.0 or citrate buffer pH6.0 (immunofluorescent analysis) for 12 min. Sections were incubated overnight with antibodies specific for Langerin (Imgenex or Novocastra), CCR6,^18,19^ CXCR7,^28^ CCL20,^18,19^ CXCL12,^28^ (R&D systems, Minneapolis, Minnesota, USA) CCR7,^18,19^ TNF (Abcam), CD31 (Neomarkers), Podoplanin (manufactured by Dr. H. Kawachi, Niigata University School of Medicine, Niigata, Japan) and/or, CXCR4,^28^ (Abnova) diluted in PBS 1% Bovine Serum Albumin. Isotype specific secondary antibodies bound to Alexa fluorochromes 488, 546 and 647 (Invitrogen) or HRP-labeled Bright-vision anti-rat-rabbit-mouse (ImmunoLogic) were applied for 30 min. Bound HRP was developed with 3,3′-Diaminobenzidine substrate (Dako). These sections were counterstained with Hematoxylin (Kinipath) and mounted with Pertex (Leica Microsystems). Immunofluorescently stained sections were mounted with homemade moviol. For a selected set of biopsies, BRAF^V600E^-mutation analysis was performed by immunohistochemistry or by real-time PCR, and in some instances with pyrosequencing, according to validated pathology protocols or according to protocols developed in research settings, including Sanquin's Research Laboratory (Amsterdam, the Netherlands).^13,15,29^ Likewise ARAF-mutations and MAP2K1-mutation analysis was performed by Whole Exome Sequencing, Sequenom and real-time PCR.^12,30^The techniques used for mutation analysis are shown per case in Table 2.

Images were recorded at 20°C by a fluorescent microscope (DM5500B, Leica Microsystems, Leica Microsystems DFC 350 FX camera, 40× original magnification) or a bright field microscope (BX41, Olympus, Olympus UC30 camera, 20× original magnification). All antibodies used in this study were validated for immunohistochemistry or immunofluorescent stainings on FFPE tissues by their manufacturers and staining results with these antibodies have been extensively reported in the literature. (Inflamed) skin and/or tonsil tissue served as positive controls; consecutive sections stained with secondary antibodies only served as negative controls.^18,28^ No immunoreactivity was detected for any type of secondary antibodies when the primary antibody was omitted (data not shown). Stained sections were scored by at least two independent researchers (WTQ, SJS, JAS or AGSH) who were blinded for clinical data as well as for BRAF-mutation status.

TNF protein expression within the lesional area was scored according to a modified Ruiter score,^31^ for staining intensity (absent (0), weak (1), clear (2) and strong (3)) and distribution (1–5% (1), 6–25% (2), 26–50% (3), 51–75% (4) and 76–100% (5)); combination scores were created by the sum of both. Langerin^+^ cells, displaying the typical round LCH-cell morphology, were scored in five representative pictures (original magnification 40×) as present or absent. Of note, LCH-affected skin biopsies often contain Langerin^+^ cells which clearly display dendrites but lack the typical round LCH-cell morphology; these cells were designated as normal epidermal LC and were excluded from the analysis.

### Flowcytometric analysis of PBMC and BMMC and chemotaxis assays

Fresh PB and BM samples were subjected to ficol density gradient centrifugation and cryopreservation in DMSO-and human albumin-enriched medium. Thawed Peripheral Blood Mononuclear Cells (PBMC) and Bone Marrow Mononuclear Cells (BMMC) were left for 1 h at 37°C, 5% CO_2_ in culture medium (Invitrogen) supplemented with 1% penicillin/streptomycin (PS) and 10% fetal calf serum (Greiner Bio-One) prior to antibody labeling or chemotaxis assays. Fresh LCH biopsies were homogenized according to a standard gentleMACS™ Dissociator protocol for processing human tumors (Miltenyi Biotec). Processed tissue was washed with ice cold PBS and filtered through a 30 μM mesh to obtain a single cell suspension.^30^ Multicolor (7 color) flowcytometric analysis was performed on a LSRII or ARIAIII flowcytometer (BD Biosciences) after labeling 1×10^4^–10^7^ PBMC or BMMC with the following antibodies: CD123-FITC, CD11c-PE, CD3/CD20/CD56-PE-Cy7, CD1a-APC, HLA-DR-V500, CXCR4-BV421 and CD14-PerCP-Cy5.5 (all from Becton Dickinson). Data were analyzed with Diva Software (BD Biosciences). Noteworthy, we have used the CD1a-specific HI149 clone and not the BB5 clone. The latter clone was initially reported to be specific for the human CD1a molecule, but it was shown later that it recognizes CD1b/c and not CD1a. Consequently, blood-born DCs described in an earlier report.^32^ were therefore incorrectly scored as being CD1a-positive. Results from a more recent report, in which the HI149 clone has been used, showed that the CD1c^+^ cells which circulate in PB have the capacity to differentiate into CD1a^+^LC.^33^ Where indicated, flow sorted CD1a^+^CD11c^+^ cells were collected in PBS/0.5% albumine for subsequent BRAF-mutation analysis by real-time PCR.^34^

For chemotaxis assays,^35^ escalating doses (0; 1; 10; 100 pg/mL) of recombinant human/rhesus macaque/feline CXCL12 (R&D systems), were added to the lower wells of a 24 ultra-low attachment plate (Costar) where after 8×10^5^ PBMC or BMMC were added to each 5.0 μm pore size trans-well insert. When indicated, the cells were pre-incubated for 15 min with 1 μg/mL of the CXCR4-antagonist AMD3100 Octahydrochloride (Sigma-Aldrich) which remained present during the total incubation period. Total cells which migrated to the lower compartment were harvested after 16 h of incubation at 37°C, 5% CO_2_ and stained with CD3-FITC, CD11c-PE, CD1a-APC (Becton Dickinson) and CD14-PerCP-Cy5.5 (PharMingen). True count beads (Cytocount, DAKO) were added in equal quantities to each condition and these mixtures were analyzed on a Calibur flowcytometer (BD Biosciences). Phenotypic characteristics and the absolute number of migrating cells per 10,000 counting beads were analyzed using BD Cell Quest Pro^TM^ Software (version 5.2.1, BD Biosciences). All conditions were tested in duplicate.^35^

### Statistical analysis

Correlation between chemokine (receptor) expression and TNF scores was tested using the non-parametric Mann–Whitney test. The influence of chemokine (receptor) expression on LCH manifestation and tissue site was analyzed using Fishers’ Exact test and on LCH reactivation rates using the Kaplan–Meier analysis, logrank test and multivariate Cox regression in SPSS version 20.0 and Graph Pad Prism version 5.0; *p* < 0.05 was considered as statistically significant.

## Results

### The majority of lesional LCH-cells express CXCR4 and/or CCR6

**Figure 1.**
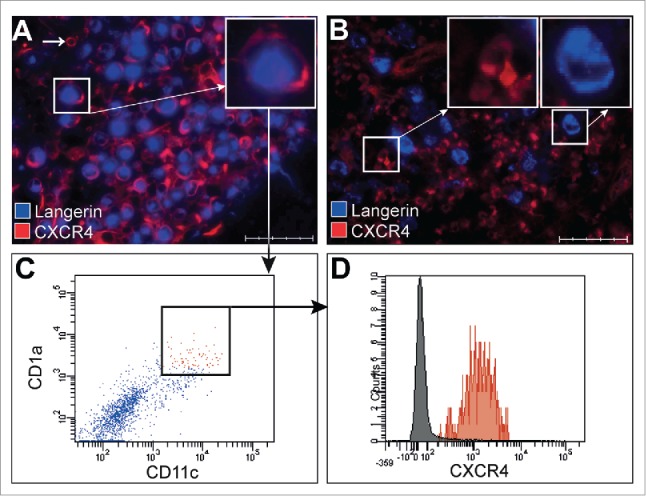
Chemokine receptor expression by LCH-cells. Representative pictures of recent onset LCH lesions subjected to triple immunofluorescent staining with antibodies directed against the LCH-cell-specific marker Langerin (CD207, blue color) in combination with the chemokine receptor CXCR4 (CD184, red color). Representative pictures were taken using a Leica Microsystems Fluorescent microscope. Original magnification 40× and scale bar defines 50 μm. Inserts depicted at the upper right corner of each photograph are a larger magnification of the indicated areas. (A) Pictures of a skin lesion from multi-system patient LCH9 showing co-localization of CXCR4 (red) on Langerin positive LCH-cells resulting in purple colored cells. Note that other cells express CXCR4 in the absence of Langerin (small white arrow in A); (B) Picture of a LN lesion showing non-LCH-cells expressing CXCR4 (left insert) and LCH-cells lacking CXCR4 visualized as single blue staining cells (right insert). (C) Representative FACS dot-plots of a 7-color based flowcytometric analysis showing gated CD1a^+^CD11c^+^ cells present in single cell suspensions prepared from a fresh LCH-affected skin biopsy from the same patient (LCH9) as depicted in Fig. 1A. (D) Representative histogram overlay showing the mean fluorescence intensity of CXCR4 expression on gated CD1a^+^CD11c^+^ cells as shown by square in Fig. 1 C  by the red histogram compared to control unlabeled cells which are shown in the gray histogram.

The two chemokine receptors most frequently expressed by Langerin^+^ LCH-cells are CXCR4 and CCR6. CXCR4 expression by LCH-cells was studied in n = 66 LCH lesions which were derived from 57 therapy-naïve patients and 4 lesions derived from 2 patients at LCH reactivation. CXCR4-positive LCH-cells were present in 46 of 66 LCH biopsies (69%, Fig. 1A) as well as in 4/4 biopsies taken at LCH reactivation. CXCR4 expression was mostly confined to bone (36/45, *p* = 0.01), but was also found in lesions taken from other anatomic locations (LN (2/4), skin (7/11) and lung (1/6). Please note that in n = 6 patients, similar CXCR4 expression was observed in different tissues taken simultaneously from the same patient. To validate the immunohistochemical staining results, we processed a fresh LCH-affected skin biopsy (LCH9) which was taken from the same location as the FFPE-biopsy shown in Fig. 1A. Mechanically dissociated CD1a^+^ LCH-cells were analyzed for CXCR4 expression by flowcytometry (Fig. 1C–1D). In both cases, CD1a^+^/Langerin^+^ LCH-cells clearly expressed CXCR4 (Fig. 1A and 1D). CXCR4 was completely absent on LCH-cells visualized in 20/66 (30%) LCH lesions (Fig. 1B). In most patients (45/57), 100% of LCH-cells either expressed or lacked CXCR4 while 12 cases showed a mixed picture in which at least 80% of the LCH-cells were positive or negative. The latter patients did not differ clinically from patients displaying homogeneous CXCR4-expression. We additionally assessed whether LCH-cells expressed other chemokine receptors involved in tissue retention (CCR6) or migration to regional lymph nodes (CCR7) in a smaller panel of LCH-affected tissues (n = 25). Serially stained sections showed differential expression of CXCR4, CCR6 and CCR7 on LCH-cells that is: CXCR4^+^ CCR6^+^CCR7^−^ (10/25), CXCR4^+^CCR6^−^CCR7^+^ (6/25), CXCR4^−^ CCR6^+^CCR7^−^ (8/25), or CXCR4^−^CCR6^−^CCR7^+^ (1/25) (data not shown).

### Lesional LCH-cells, stromal cells and vessels express the CXCR4 ligand CXCL12

**Figure 2.**
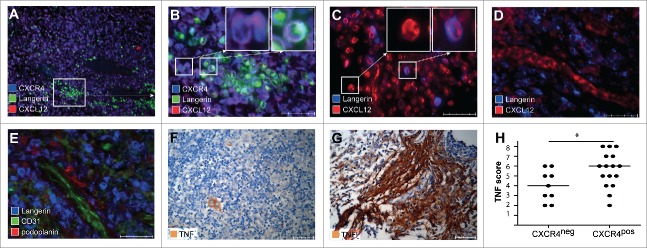
High TNF and CXCL12 expression in LCH lesions correlates with the presence of CXCR4^+^ LCH-cells. LCH lesions (n = 25) were stained with antibodies specific for the LCH-cell-specific marker Langerin (green color in A–B and blue color in C–E), CXCL12 (red color in A–D),CXCR4 (blue color in A–B), CD31 (green color in E) and Podoplanin (red color in E) or with TNF (brown color in F–G) Representative pictures are taken from lesions with different distribution of CXCL12 (original magnification 40×, scale bar defines 50μm) and of TNF original magnification 20×, scale bar defines 100 μm). Inserts depicted at the upper right corner of B and C are a larger magnification of the cells in the indicated areas. (A–B) Pictures taken at 10× magnification (A) and 40× magnification (B) from the depicted area in A. Pictures are showing that CXCL12 is expressed by CXCR4^+^ Langerin^+^ LCH-cells, resulting in a purple, turquoise cell (right insert in B) which are surrounded by Langerin^−^ bystander cells that co-express CXCL12 and CXCR4 (left insert in B) (C) Picture showing that CXCL12 is expressed by Langerin-negative cells (left insert) and by Langerin-positive LCH-cells, resulting in a purple cell (right insert). (D) Picture demonstrating that CXCL12 is expressed at endothelial cells lining vessels (red) which are surrounded by Langerin-positive-LCH-cells (blue color). (E) A picture taken from the same location in a serial section prepared from the same LCH lesion as displayed in B. This photograph shows that LCH-cells (blue color) surround CD31^+^ (green color) blood vessels, while some cells stain positive for Podoplanin (red color). (F) Picture of a representative LCH lesion with a TNF combination score three (moderate staining intensity and expression of less than 5% of cells present in the biopsy) and (G) Picture of a representative LCH lesion with combination score eight (strong staining intensity and expression of more than 75% of the cells present in the biopsy) (H) Graph showing correlation analysis of the combination scores of TNF and CXCR4 expression by LCH-cells within the same lesion. Lesions with high TNF scores contained more CXCR4^+^ LCH-cells (*p* = 0.01). Line represents median TNF score.

Based on the finding that the majority of LCH-cells express CXCR4, we assessed the *in situ* expression of its ligand CXCL12 (Fig. 2). Due to limited biopsy material we randomly excluded some samples from CXCL12 analysis. Langerin^+^ LCH-cells expressed CXCL12 in the majority (39/66) of LCH biopsies taken at diagnosis (Fig. 2A-2C). In these 39 biopsies, the CXCL12 expression occurred either in the presence (34/39, Fig. 2A and 2B) or absence (5/39) of CXCR4 expression (data not shown). In addition, none of the CXCR4-negative CXCL12^+^ LCH-cells co-expressed CXCR7, the alternative chemokine receptor which binds CXCL12 (data not shown).^36^ In 55 of 57 patients, CXCL12 was abundantly expressed in the micro-environment of the lesion as well as in the surrounding tissue by Langerin-negative, presumably stromal cells (single purple cells in Fig. 2A and 2B and red cells depicted in Fig. 2C), and by endothelial cells lining microvasculatures (Fig. 2D). The differential expression pattern of podoplanin, which is exclusively expressed by lymphatic vessels, and CD31, which is mainly expressed by blood vessels, discriminates these vasculatures.^37^ Additional Immunofluorescent staining experiments revealed that Langerin^+^ LCH-cells are located around CD31^+^ blood vessels in the co-presence of some podoplanin^+^ cells (Fig. 2D and 2E). Taken together, CXCL12 was thus detected in 68/70 (97%) biopsies.

### The presence of CXCR4-positive LCH-cells correlates with the extent of locally produced TNF

We next investigated whether TNF, a known inducer of CXCR4 and CXCL12 under inflammatory conditions,^21,24^ is expressed in LCH lesions. TNF expression varied from weak to strong staining intensity; likewise, the percentage of positively staining cells ranged from just a few cells (Fig. 2F) to the vast majority of cells present in the lesions being positive (Fig. 2G). Although the presence of CXCL12^+^ LCH-cells did not correlate with TNF score (*p* = 0.18, data not shown), lesions containing CXCR4^+^ LCH-cells had a significantly higher mean TNF score.(*p* = 0.01, Fig. 2H)

### Detection of CXCL12-responsive CD1a^+^CXCR4^+^ cells in peripheral blood and bone marrow samples derived from LCH-patients with active disease

**Figure 3.**
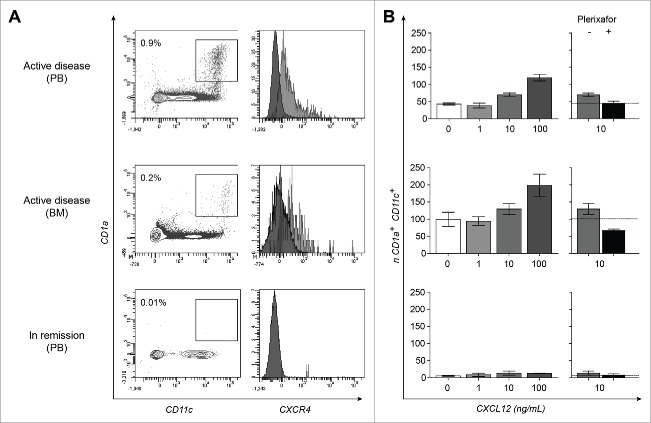
CXCL12-responsive CD1a^+^CXCR4^+^ cells are present in PB and BM samples collected during active multi-system LCH manifestation. Representative FACS dot-plots of a seven-color based flowcytometric analysis (A) and graphs from migration assays (B) performed with PBMC and BMMC from a multi-system patient (LCH7 analyzed at disease onset (upper and middle row respectively) and PBMC collected 5 y after recovery (lower row)). (A) Dot-plot of PBMC showing the increased percentage of Lin^−^(CD3^−^CD20^−^CD56^−^) CD14^+^HLA-DR^+^CD1a^+^CD11c^+^ cells (cells depicted in the box) and the mean fluorescence intensity of CXCR4 expression by these CD1a^+^CD11c^+^ cells (gray histogram in right plot) compared to unlabeled cells (black histogram in right plot) are shown. Percentage represents the number of CD1a^+^CD11c^+^ and CD1a^+^CD14^+^ cells among the Lin^−^(CD3^−^CD20^−^CD56^−^) HLA-DR^+^ population within the indicated box. (B) Left graphs show the number of CD1a^+^CD11c^+^ cells per 10,000 true count beads which migrated in trans well plates from the upper compartment to the lower compartment containing escalating doses of the chemo attractant CXCL12 (0; 1; 10; 100 pg/mL) as measured by flowcytometry. The corresponding right graphs show the migration of Lin^−^CD1a^+^CD11c^+^ cells toward 10 ng/mL CXC12 in the presence (black bars) or absence (gray bars) of the CXCR4-blocking reagent AMD3100 (Plerixafor®), which completely reduced the migration to basal levels in the absence of CXCL12 (horizontal lines). Data represents the mean number of migrating cells per condition as measured in duplicate.

Based on our *in situ* observations, we hypothesized that CXCR4^+^ LCH-cells are actively recruited from the circulation into CXCL12-expressing tissues. We therefore searched for the presence of CD1a and CXCR4 co-expressing cells in PBMC (n = 17) and BMMC (n = 5) derived from 13 therapy-naive LCH-patients with different clinical manifestation forms (3 multi-system, 4 poly-ostotic, 5 mono-ostotic and 1 diabetes insipidus); samples were collected at different time points that is, at onset (n = 14, one PBMC and one BMMC were collected from the same patient), after initiating any form of treatment (n = 2), after reaching non-active disease (n = 4) or at LCH reactivation (one PBMC and one BMMC sample from the same patient). In 5 out of 14 samples collected from therapy-naive patients with multi-system LCH (n = 3) or with mono-ostotic (n = 1) LCH, a distinct population of CD1a^+^ cells within the Lin^−^ (CD3^−^CD20^−^CD56^−^) HLA-DR^+^ cells (varying from 0.01% to 0.6% in PBMC and 0.04%–0.2% in BMMC) was detected. This population could be detected in both PBMC and BMMC from one multi-system patient (Fig. 3A and 3B). Whereas CD1a^+^ cells co expressed CD11c and CXCR4 in all four patients, co expression of CD14 was seen in all but one patient (Fig. S1 for the corresponding gating strategy). These ‘LCH-like’ cells with myeloid features were not detected when BMMC and/or PBMC were collected from the same patients after initiation of systemic chemotherapy or after therapy-induced remission; no follow-up samples were available from the other two patients with circulating CD1a^+^ cells. CD1a^+^CXCR4^+^ cells were neither detected in samples collected from the other therapy-naive patients (n = 9), nor from patients in remission (n = 5) nor in healthy controls (n = 8) (data not shown). Note that all patients with circulating HLA-DR^+^CD1a^+^CD11c^+^CXCR4^+^ cells also displayed CXCR4^+^ LCH-cells *in situ* at LCH onset as is shown in Fig. 1A for LCH9.

Mutation analysis was performed on biopsied tissue samples and circulating LCH-like cells obtained from these four patients. While patient LCH8 displayed BRAF^wild type^/MAP2K1^mutated,^^12^ LCH-cells in his tissue biopsy, flow-sorted ‘LCH-like’ cells also expressed the BRAF^wild type^ DNA sequence (data not shown); Note that MAP2K1 analysis could not be performed due to limited DNA yields. Likewise, the DNA extracted from flow-sorted CD1a^+^ cells of the two patients in whom the BRAF^V600E^ mutation was identified in biopsied tissue specimens (LCH6 and LCH9, Table 2) did not yield enough template for reliable BRAF analysis. The BRAF^V600E^ DNA sequence was however detected in DNA extracted from unseparated BM cells obtained from patient LCH9 (data not shown).

Next, the chemotactic capacity of circulating CD1a^+^CD11c^+^CXCR4^+^ cells toward CXCL12 was addressed using a standard trans-well chemotaxis assay.^35^ To this end, we selected the patient who presented with the highest percentage of circulating CD1a^+^ cells at disease onset. Only during active disease, CD1a^+^CD11c^+^ cells present in PBMC and BMMC migrated to CXCL12 in a dose-dependent manner (Fig. 3B). When PBMC were collected from the same patient after LCH recovery, only very few CD1a^+^CD11c^+^ cells migrated to CXCL12 (Fig. 3B), similarly to PBMC from healthy controls (data not shown). The active migration of CD1a^+^CD11c^+^ cells toward CXCL12 was effectively reduced to baseline migration after pre-exposure of PBMC or BMMC to the CXCR4 inhibitor Plerixafor®).

### LCH-patients with CXCR4-positive LCH-cells at diagnosis are highly prone to develop LCH at multiple sites and to reactivate

**Figure 4.**
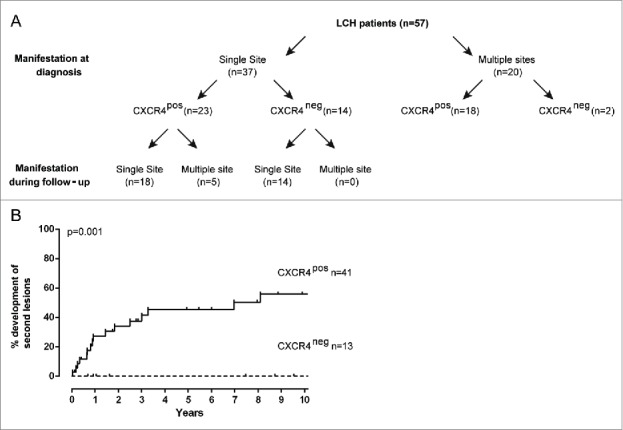
Patients displaying CXCR4^+^ LCH-cells at diagnosis are more likely to develop LCH at multiple sites and are prone to LCH reactivation. (A) Flow diagram showing the association between CXCR4 expression on LCH-cells in primary LCH lesions with the manifestation of LCH either at a single or at multiple sites at diagnosis (upper row) or during the entire follow-up period (lower row). Note that, poly-ostotic lesions and LCH lesions in multiple organ systems were collectively designated as ‘LCH manifestations at multiple sites’; mono-ostotic lesions and solitary skin, lung or LN lesions were designated as ‘single site LCH manifestation’. Follow-up data were not available from two patients with single-site disease; these patients were designated as single-site lesions in follow-up. (B) Kaplan–Meier analysis showing that none of the 13 of LCH patients in whom the LCH-cells lacked membrane CXCR4 expression reactivated within 10 y after the primary diagnosis. On the contrary, nearly half of the patients (17/41) with CXCR4^+^ LCH-cells at disease onset showed LCH reactivation later in time. Clinical follow-up was incomplete for five patients, who were excluded from the correlation analysis. Clinical follow-up for nine patients was longer than ten years without any event.

Based on our observations that intralesional blood vessels express CXCL12 and CXCL12-responsive CXCR4^+^ myeloid cells are circulating during active disease, we retrospectively investigated the clinical relevance of CXCR4 expression by lesional LCH-cells in relation to primary LCH manifestation and disease progression (Fig. 4A). CXCR4-negative-LCH-cells were detected in 14 patients who presented with a single LCH lesion and their lesion resolved during follow-up. In contrast, 5/23 patients (22%) who initially presented with a single-site lesion containing CXCR4^+^ LCH-cells developed multiple LCH lesions during follow-up. In the group of patients who presented with LCH at multiple sites (defined as multi-system LCH with and without risk-organ involvement or poly-ostotic LCH), 18/20 patients (90%) displayed CXCR4^+^ LCH cells at diagnosis (Fig. 4A); 4/6 (66%) poly-ostotic LCH patients in the latter group developed a multi-system variant of LCH during follow-up. The two exceptional patients who presented with multiple LCH lesions containing CXCR4-negative LCH-cells were (1) an 11 y old girl with poly-ostotic LCH who achieved complete remission after chemotherapy and did not show any signs of reactivation within 10 y of follow-up and (2) a 56 y old male who presented with a disseminated form of LCH with poly-ostotic lesions, involvement of LN and possible involvement of lung and liver. As he was also diagnosed with metastasized urothelial cell carcinoma and prostate carcinoma no anti-LCH treatment was initiated and he was lost for follow-up after 2 y.

CXCR4 expression correlated either with primary LCH manifestation at multiple sites (Fisher's Exact Test *p* = 0.02) as well as with the development of multiple lesions during follow-up (*p* = 0.003). In addition, biopsies from 4/4 patients with poly-ostotic or multi-system involvement collected from multiple sites respectively at diagnosis and at reactivation all contained CXCR4^+^ LCH-cells.

Subsequent Kaplan–Meier analysis revealed that 17/41 (44%) of the patients who presented with CXCR4^+^ LCH-cells at LCH onset developed secondary lesions within 10 y after diagnosis while none of the 13 patients with CXCR4-negative LCH-cells reactivated (log-rank test *p* = 0.001, sensitivity of 100%, specificity of 35%), Fig. 4B). In addition, while disease manifestation (both disseminated disease as well as multiple lesions at diagnosis) and BRAF mutation status were risk factors for reactivation (log-rank test *p* = 0.02, *p* = 0.02, *p* = 0.05), age and TNF expression were not a risk factor (log-rank-test *p* = 0.71, *p* = 0.84, respectively). However, CXCR4 status at LCH onset was shown to be an independent risk factor for reactivation when corrected for age (odds ratio 8.7; *p* = 0.013) the presence of multiple lesions at diagnosis (odds ratio 12.2; *p* = 0.002), BRAF mutation status (odds ratio 10.1; *p* = 0.006) and TNF expression (odds ratio 7.1; *p* = 0.02) as well as corrected for age, manifestation at diagnosis and BRAF mutation status (odds ratio 10.45; *p* = 0.03). Note that follow-up data from three patients who presented with CXCR4-negative LCH-cells at diagnosis are missing. Moreover, clinical data on the involvement of risk organs are missing for the vast majority of patients. Hence, whether the involvement of risk organs is a risk factor for reactivation in this cohort cannot be analyzed. These results indicate that in this randomly selected patient cohort the presence of CXCR4-positive LCH-cells at primary LCH manifestation has a negative impact on the patients’ clinical outcome.

## Discussion

The unpredictable outcome of LCH, including disease reactivation, the unusual homing characteristics and presumed myeloid origin of the aberrantly differentiated LCH-cells have all been studied independently. Given their pleiotropic functions, chemokines likely play a role in the maintenance and/or development of LCH predilection sites, as well as in directing LCH lesion infiltration of other immune cells (arrow in Fig. 1A), all reminiscent signs of chronic inflammation. In this study, CXCR4 expression by LCH-cells was found to be associated with multiple site LCH manifestation and elevated risk of disease reactivation regardless of the mutation status of LCH-cells at disease onset. In contrast, as the sensitivity of CXCR4 assessment in relation to reactivation is 100% in this sample set, patients who's LCH-cells lack CXCR4 have a very limited risk to reactivate. Whereas CCR6 seems to be required for retention of LCH-cells in the various tissues, our *in vitro* experiments support a role for CXCR4 in the distribution of blood-borne CD1a^+^CD11c^+^CXCR4^+^‘LCH-cell like’ myeloid cells in analogy to both immune cells and tumor cells.^21^

Given the similarity in CXCR4 gene expression profiles of LCH-cells isolated from multi-and single-site patients,^38,39^ our immunohistochemistry findings point out that visualization of protein expression by LCH-cells remains an essential step to confirm the functional relevance of their mRNA signature. Discrepancies between mRNA and protein expression levels may indicate that posttranscriptional processes, among others, can influence the production level, stability and recycling pattern of membrane-expressed proteins. Furthermore, the balance between pro-and anti-inflammatory cytokines, like TNF produced by other lesional cells, may have an exogenous effect on CXCR4 protein expression by LCH-cells.^40,41,42^ As the presence of CXCR4^+^ LCH-cells correlated to the extent of TNF produced in the lesion, we hypothesize that TNF plays a role in stabilizing cell surface expression of CXCR4 by LCH-cells which subsequently controls their CXCL12-responsiveness. As reactivation rates were solely influenced by the presence of CXCR4 and not by intra-lesional TNF levels (high versus moderate to low), CXCR4 is probably not a surrogate marker for high TNF levels co-present in the lesion. Elevated TNF serum levels.^43^ may, however, stabilize CXCR4 expression on the circulating CD1a^+^CXCR4^+^cells which were visualized by multicolor flowcytometric analysis of PB or BM samples collected at LCH onset from four patients with active LCH (Table 2). Increased plasma levels of TNF as well as TNF-induced cytokines and chemokines were also observed in patients with ECD.^44^ However , the relevance of high TNF levels in ECD is unclear as it is unknown whether (1) ECD-associated histiocytes express CXCR4 *in situ*, (2) aberrant histiocytes circulate in these patients and (3) TNF could induce CXCR4 on these cells. Given that LCH lesions are heavily vascularised and that these blood vessels were found to express CXCL12, we speculate that CXCR4 expression supports both the entry of BM-derived LCH-cell precursors via the bloodstream to CXCL12-positive tissues and the re-direction of LCH-cells which arise as a consequence of somatic mutations *in situ*.^6^ In accordance with the already defined phenotype of lesional LCH-cells,^45-47^ majority of the circulating CD1a^+^ cells reported in this study clearly co-expressed HLA-DR, CD11c and CD14. Increased percentages of myeloid CD11c^+^ cells in PB collected from LCH-patients were earlier reported, however, both studies never performed CD1a nor Langerin co-labeling.^6,48^ The recently published finding that genetically modified mice expressing BRAF^V600E^-alleles under the CD11c-promoter develop LCH-like lesions,^6^ implies that CD1a^+^CD11c^+^ cells are possibly the direct precursors of LCH-cells found *in situ*. This may explain why the genetic signature of tissue-isolated LCH-cells is significantly different to that of PB-derived monocytes, plasmacytoid and myeloid DC.^39^ Our findings extend the recent finding that circulating BRAF^V600E^ expressing CD11c^+^ and CD14^+^ cells are only present in PB and BM from patients with multiple lesions.^6^ While CD1a^+^CD11c^+^CD14^+^CXCR4^+^ cells were detected in patients expressing BRAF^V600E^ LCH-cells *in situ* (LCH6 and LCH9), we here report that circulating CD1a^+^CD11c^+^CD14^+^CXCR4^+^ cells were also detected in a patient who had lesional LCH-cells expressing a MAP2K1-mutation (LCH8).^12^ as well as in a patient with BRAF^wild type^ lesional LCH-cells (LCH7). Through extending the number of antibodies for prospective screening of PB or BM samples from newly onset LCH patients, we expect to be able to finally answer the question whether these circulating CD1a^+^ ‘LCH-like’ myeloid cells are actually mature CD207^+^ LCH-cells which have re-entered the circulation or whether these cells represent the long sought BM-derived precursor cells which give rise to tissue-resident LCH cells as suggested by Berres *et al.*.^6^ As LCH is a rare disease it will take some time to collect enough samples to confirm the prospective value of these circulating cells.

The only low-risk multi-system patient in our study who lacked circulating CD1a^+^ cells at disease onset displayed a heterogeneous expression pattern of CXCR4 *in situ* in comparison to the other three multi-system patients in whom circulating CD1a^+^ cells could be clearly visualized. It is tempting to speculate that the LCH-cells in this patient have acquired CXCR4 expression after entering the tissue. CXCL12 binding to CXCR4 on monocyte-derived dendritic cells induces ERK1/2 phosphorylation which subsequently leads to increased proliferation and survival of these cells.^23^ As shown by data from the Rollins group,^13^ LCH-cells, on the contrary, express constitutively high-levels of phospho-ERK1/2 regardless of their BRAF mutation status. Given that CXCL12 is expressed in both an autocrine and paracrine fashion, we postulate that local CXCR4-CXCL12 signaling leads to increased proliferation and survival of tissue-resident LCH-cells through providing anti-apoptotic signals *in situ*. While BRAF^V600E^ and CXCR4 co-expressing LCH-cells were found in 13/14 (93%) biopsies, 13/19 (68%) BRAF^wild type^ LCH-cells also expressed CXCR4 (Tables 1 and 2). It remains to be studied whether signaling through CXCR4 alone is sufficient to induce constitutive MAPK activation,^13^ in the absence of known somatic mutations like the recently described ARAF or MAP2K1 mutations,^10-12,30^ The retrospective nature of our study refrained us from testing this hypothesis. Given the observed CXCL12 responsiveness of circulating CD1a^+^CD11c^+^cells (Fig. 3B), it would also be of interest to use the recently developed BRAFV600E^CD11c^ mouse model to study CXCR4 expression in the myeloid compartment of these mice and the therapeutic action of the FDA-approved CXCR4 antagonist AMD3100,^49^ on disease manifestation and progression in these mice.

In conclusion, this study provides the first evidence that *in situ* CXCR4 expression at primary LCH manifestation is an independent prognostic marker for disease progression and reactivation. Whether patients who's LCH-cells lack CXCR4 indeed have a limited risk to reactivate, is currently addressed in a prospective add on study to LCH-IV which is performed in collaboration with the Dutch Childhood Oncology Group.
